# Physics Informed Neural Networks (PINN) for Low Snr Magnetic Resonance Electrical Properties Tomography (MREPT)

**DOI:** 10.3390/diagnostics12112627

**Published:** 2022-10-29

**Authors:** Adan Jafet Garcia Inda, Shao Ying Huang, Nevrez İmamoğlu, Ruian Qin, Tianyi Yang, Tiao Chen, Zilong Yuan, Wenwei Yu

**Affiliations:** 1 Department of Medical Engineering, Chiba University, Chiba 263-8522, Japan; 2 Department of Surgery, National University of Singapore, Singapore 119077, Singapore; 3 Engineering Product Development Department, Singapore University of Technology and Design, Singapore 487372, Singapore; 4 Digital Architecture Research Center, National Institute of Advanced Industrial Science and Technology, Tokyo 135-0064, Japan; 5 Department of Radiology, Hubei Cancer Hospital, Tongji Medical College, Huazhong University of Science and Technology, Wuhan 430079, China; 6 Center for Frontier Medical Engineering, Chiba University, Chiba 263-8522, Japan

**Keywords:** MREPT, machine learning, neural network, physics informed neural network, PINN

## Abstract

Electrical properties (EPs) of tissues facilitate early detection of cancerous tissues. Magnetic resonance electrical properties tomography (MREPT) is a technique to non-invasively probe the EPs of tissues from MRI measurements. Most MREPT methods rely on numerical differentiation (ND) to solve partial differential Equations (PDEs) to reconstruct the EPs. However, they are not practical for clinical data because ND is noise sensitive and the MRI measurements for MREPT are noisy in nature. Recently, Physics informed neural networks (PINNs) have been introduced to solve PDEs by substituting ND with automatic differentiation (AD). To the best of our knowledge, it has not been applied to MREPT due to the challenges in using PINN on MREPT as (i) a PINN requires part of ground-truth EPs as collocation points to optimize the network’s AD, (ii) the noisy input data disrupts the optimization of PINNs despite the noise-filtering nature of NNs and additional denoising processes. In this work, we propose a PINN-MREPT model based on a canonical analytic MREPT model. A reference padding layer with known EPs was added to surround the region of interest for providing additive collocation points. Moreover, an optimizable diffusion coefficient was embedded in the analytic MREPT model used in the PINN-MREPT. The noise robustness of the proposed PINN-MREPT for single-sample reconstruction was tested by using numerical phantoms of human brain with extra tumor-like tissues at different noise levels. The results of numerical experiments show that PINN-MREPT outperforms two typical numerical MREPT methods in terms of reconstruction accuracy, sensitivity to the extra tissues, and the correlations of line profiles in the regions of interest. The advantage of the PINN-MREPT is shown by the results of an experiment on phantom measurement, too. Moreover, it is found that the diffusion term plays an important role to achieve a noise-robust PINN-MREPT. This is an important step moving forward to a clinical application of MREPT.

## 1. Introduction

Electrical properties (EPs) of biological tissues (permittivity, ϵ, and conductivity, σ) are quantitative properties that could facilitate the identification of non-calcified cancerous tissues [1,2,3,4]. Moreover, the knowledge of the EP distribution of human tissues is a base to calculate the specific absorption rate (SAR) for RF safety when humans are exposed to RF fields, e.g., wireless power transfer [5], ultra-high field MRI [6]. There are in vivo methods to measure EPs of human tissues, such as electrical impedance tomography (EIT) [1], magnetic induction tomography (MIT) [7], magnetic resonance electrical impedance tomography (MREIT) [8], and magneto-acoustic tomography with magnetic induction (MAT-MI) [9]. However, these approaches require either stand-alone apparatuses [1,7,9] or apparatuses added to a magnetic resonance imaging (MRI) scanner [10].

In parallel to the techniques above, magnetic resonance electrical properties tomography (MREPT) is a technique to non-invasively reconstruct EPs of tissues from MRI measurements (i.e., the measurable RF fields called B${}_{1}$-fields [11]) without additional apparatuses [12,13]. The canonical formulation of MREPT is derived from Maxwell’s equations [14]. The reconstruction of the EPs relies on the measurement of the magnitude and phase of the B${}_{1}$-fields, $B_{1}^{+}=\left|B_{1}^{+}\right|e^{-i\varphi^{+}}$ for transmit fields and $B_{1}^{-}=\left|B_{1}^{-}\right|e^{i\varphi^{-}}$ for receive fields [15]. Initially in [14], the reconstruction of the complex permittivity, $\kappa =\sigma /\omega \epsilon_{0}+i\epsilon_{r}$, was focused on (ω is the angular frequency).

To shorten scan time, conductivity-focused formulations were developed [16], in which, the B${}_{1}$ phase sum, $\varphi^{\mathrm{tr}}=\varphi^{+}$ + $\varphi^{-}$, also known as the transceive phase, is used to reconstruct the conductivity of tissues. The phase of $B_{1}^{+}$ ($\varphi^{+}$), is estimated using the half phase assumption [14], i.e., $\varphi^{+}\approx \varphi^{\mathrm{tr}}/2$. The approximation holds when the field strength (B${}_{0}$) is low [17].

In the clinical environment, the measurements of $\varphi^{\mathrm{tr}}$ are contaminated with noise, which results in artifacts in the MREPT conductivity reconstructions, especially when numerical differentiation (ND) is needed [18,19]. Hence, MREPT can benefit from any method that can reduce either the noise of $\varphi^{\mathrm{tr}}$ measurements or the errors caused by the ND of $\varphi^{\mathrm{tr}}$ contaminated with noise.

On the other hand, neural networks (NNs), graphs composed of various layers of parameters optimized to mimic a function from data sample pairs, have been proposed for many image reconstruction methods [20]. NNs have been actively applied to MREPT in EP reconstruction and $\varphi^{\mathrm{tr}}$ denoising. In [21], an end-to-end NN reconstruction of EPs from $B_{1}$-fields was proposed. The training was done without any prior knowledge of physics embedded in analytic models. For denoising, a noise filtering method for the $B_{1}$-fields measurements was proposed, where a NN black-box model was learned from numerically simulated samples for filtering the $\varphi^{\mathrm{tr}}$ [22]. Although it shows improved accuracy for numerically simulated samples, it lacks generalization or robustness for its clinical uses [23]. Recently, we proposed physics-coupled NNs where the coefficients in the MREPT formulation are optimized and the physics are embedded into the NNs [24]. Specifically, the values of global or pixel-wise coefficients to control a viscosity-type regularization [25] on a convection-reaction-diffusion MREPT formulation [16] were optimized through a NN by a differentiable physics model [26], i.e., coupling physical knowledge to the NN’s optimization to improve the reconstruction robustness. However, same as the others, this method also relies on learning from datasets of numerical simulations. Although the physical knowledge reduces the size of the dataset needed for robust reconstruction, the size of the dataset necessary for clinical robustness is still large [24].

As a matter of fact, big clinical datasets for training the NNs for MREPT are not available. Therefore, the datasets for training have been constructed from numerically simulated samples [21,22,24,27]. While simulated datasets are appropriate for many applications, there are limitations of such datasets in the medical image reconstruction field [28]. Specifically, when the simulated datasets are applied, the resultant reconstruction may suffer from a lack of generalization or robustness [23], because measurement-related effects, noise sources, or tissue structures that are unaccounted for during the learning procedure may affect it. Hence, methods that can reduce the number of samples for training or avoid the dependency on a training dataset are necessary for the clinical uses of MREPT.

Recently, physics-informed NNs (PINNs) were introduced to solve partial differential Equations (PDEs) [29]. PINN is a technique where a NN is trained to map the spatial coordinates to a field of interest, i.e., to map the independent variables to a dependent variable according to prior knowledge such as boundary conditions, and/or collocations points of the dependent variables according to a fitting loss function. Using NNs automatic differentiation (AD) through backpropagation [30], the spatial partial derivatives with respect to the predicted dependent variables are calculated. Then, the spatial partial derivatives are used to solve a PDE. This induces the NN to map the function of interest and enforce the solution of the PDE. In summary, PINNs are used to predict a field of interest from spatial coordinates, by using the residual of the PDE to codify information from areas non-covered by collocation points or boundary conditions. This method has been used to solve highly complex PDEs (many dependent variables) [31], to increase the speed of the solution of a PDE while maintaining the accuracy of numerical methods [29] and to recover or discover parameter values in design applications [32].

It is promising to apply PINN to MREPT based on the advantages associated with NN’s AD [33]. However, it is worth noting that PINN has limitations. First, collocation points of the dependent variable are needed to optimize the PINN. Second, in cases where the physical model is not fully known and inputs contain high level of noise, the optimization of the PINN is compromised. These limitations can become more prominent when PINN is applied to move MREPT forward toward its clinical uses. First, it is difficult to assume that the EPs of part of the tissues of interest are known for most diagnostic cases. Second, unavoidable noise contamination associated with $\varphi^{\mathrm{tr}}$ will disrupt PINN-MREPT’s optimization process.

In this work, we propose a PINN-MREPT model based on a canonical analytic MREPT model. First, a reference padding layer with a specific known EP was added to surround the region of interest for providing additive collocation points. Second, a dual approach was taken to reduce the noise: (1) to use the NN as a low pass filter [34] to predict $\varphi^{\mathrm{tr}}$. (2) to avoid the noise explosion of ND during optimization, the spatial partial derivatives of $\varphi^{\mathrm{tr}}$ are calculated through the NN’s AD, and $\hat{\sigma }$ is calculated by an optimizable differentiable physics MREPT formulation [24].

Comparisons between proposed PINN-MREPT with numerical analytic models were made by experiments on numerical simulations of digital human phantoms. Detailed descriptions of the formulations, numerical simulations, and the PINN-MREPT method are as follows.

## 2. Materials and Methods

### 2.1. MREPT Formulations

A canonical formulation for phase-based conductivity reconstruction is standard-EPT (std-EPT) [14]. In std-EPT, a homogeneity assumption (∇γ=0), i.e., the conductivity is assumed to be constant, is ascertained for the reconstructed conductivity (σ) [14]. The formulation is shown below:

(1) $$\gamma =\frac{2\omega \mu_{0}}{\nabla^{2}\varphi^{\mathrm{tr}}}$$

where γ is the inverse of the conductivity (γ=1/σ), $\mu_{0}$ is the magnetic permeability, which is considered to be the permeability of free space in the tissues under study. However, the homogeneity assumption for the std-EPT formulation results in artifacts near tissue boundaries [18]. To counteract the boundary artifacts, a formulation without the homogeneity assumption was proposed, convection-reaction EPT (cr-EPT) [35].

In cr-EPT, the ∇γ-term, i.e., the gradient of conductivity, is maintained as an unknown, and the resulting PDE is discretized and solved as a set of linear equations. A corresponding phase-based cr-EPT formulation was proposed [16]. It is shown as follows:

(2) $$\nabla \varphi^{\mathrm{tr}}\cdot \nabla \gamma +\nabla^{2}\varphi^{\mathrm{tr}}\gamma =2\omega \mu_{0}$$

However, the solution of this PDE results in spurious numerical errors throughout the image. This effect can be reduced as the discretization mesh is refined [36], however, this will increase the required scan time and the reconstruction’s computational cost. To improve the stability of the solution of the PDE, a diffusion term that acts as a viscosity regularization term on the solution, $\nabla^{2}\gamma$, can be added to the formulation to dampen the spurious numerical errors [25]. However, the viscosity regularization term can reduce the contrast of the σ reconstruction. Thus, a diffusion coefficient is helpful to regulate the diffusion term, leading to a revised formulation as,

(3) $$\nabla \varphi^{\mathrm{tr}}\cdot \nabla \gamma +\nabla^{2}\varphi^{\mathrm{tr}}\gamma -\rho \nabla^{2}\gamma =2\omega \mu_{0}$$

where ρ is the diffusion coefficient. This coefficient is usually selected empirically and added homogeneously [25,37] because there are no explicit rules on the decision of the diffusion coefficient. Numerous follow-up investigations on the selection and optimization of the formulation in Equation (3) were reported [24,37,38,39]. Equation (3) is a PDE that is solved by discretizing the region of interest and applying central differences. The discretized formulation is shown as follows,

(4) $$\begin{array}{c} 2\omega \mu_{0}=\gamma_{i,j}\left[\frac{\partial^{2}\varphi_{x}^{\mathrm{tr}}}{\partial x^{2}}+\frac{\partial^{2}\varphi_{y}^{\mathrm{tr}}}{\partial y^{2}}\right]\\ +\left[\frac{\partial \varphi_{x}^{\mathrm{tr}}\left(\gamma_{i+1,j}-\gamma_{i-1,j}\right)}{2\partial x}+\frac{\partial \varphi_{y}^{\mathrm{tr}}\left(\gamma_{i,j+1}-\gamma_{i,j-1}\right)}{2\partial y}\right]\\ -\rho \left[\frac{\gamma_{i+1,j}-2\gamma_{i,j}+\gamma_{i-1,j}}{\partial x^{2}}+\frac{\gamma_{i,j+1}-2\gamma_{i,j}+\gamma_{i,j-1}}{\partial y^{2}}\right] \end{array}$$

where *i* and *j* are the index number for pixels in the x- and the y-direction, respectively. The set of linear equations is interpreted as an Ax=b system where the values of $\gamma_{i,j}$ lie in the unknown array, x.

### 2.2. PINN-MREPT

**Figure 1:**
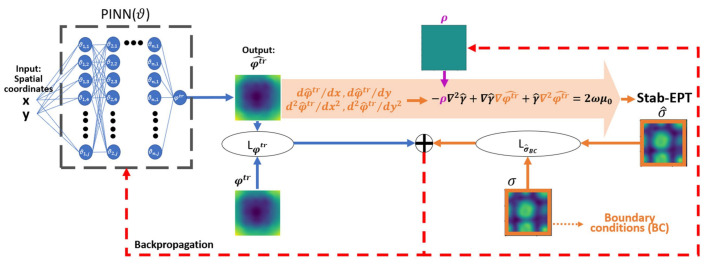
The flow of the proposed PINN-MREPT for the reconstruction of conductivity (σ) based on the stab-EPT formulation. The PINN on the left predicts the phase (${\hat{\varphi }}^{\mathrm{tr}}$) from the spatial coordinates (x,y) and calculates its difference to the true grand phase, i.e., the loss term ($L_{\varphi^{\mathrm{tr}}}$). Then, the spatial partial derivatives of the phase ($\nabla {\hat{\varphi }}^{\mathrm{tr}},\nabla^{2}{\hat{\varphi }}^{\mathrm{tr}}$) are calculated by NN’s AD. The predicted $\nabla {\hat{\varphi }}^{\mathrm{tr}},\nabla^{2}{\hat{\varphi }}^{\mathrm{tr}}$ are then used to solve the stab-EPT formulation according to the discretization shown in Equation (4) to predict $\hat{\sigma }$ and calculate its difference from the known conductivity σ of the reference padding, i.e., the loss term ($L_{{\hat{\sigma }}_{\mathrm{BC}}}$). The losses are summed and backpropagated to modify the PINN parameters (ϑ) and the diffusion coefficient of the stab-EPT formulation (ρ).

The flow of the proposed PINN-MREPT based on the stab-EPT formulation is shown in Figure 1. As shown at the left of Figure 1, the NN receives the spatial coordinates (x,y) as inputs, and outputs a predicted value of $\varphi^{\mathrm{tr}}$ in the location indicated by the input spatial coordinates, denoted as ${\hat{\varphi }}^{\mathrm{tr}}$(x,y). At the output of the NN, as shown by the downward arrows in the middle of Figure 1, a loss function is defined by comparing the predicted ${\hat{\varphi }}^{\mathrm{tr}}$ and the measured $\varphi^{\mathrm{tr}}$ (ground truth) as follows,

(5) $$L_{\varphi^{\mathrm{tr}}}=L_{MSE}\left(\varphi^{\mathrm{tr}},{\hat{\varphi }}^{\mathrm{tr}}\right)+L_{SSIM}\left(\varphi^{\mathrm{tr}},{\hat{\varphi }}^{\mathrm{tr}}\right)$$

where $L_{MSE}$ is the mean square error and $L_{SSIM}$ indicates the loss based on the structural similarity index (SSIM) [40] in its minimization form (1-SSIM). The combination of MSE and SSIM has been selected to balance the losses reflecting local accuracy (MSE) and global accuracy (SSIM) in the reconstructions [41] The output from the PINN, the predicted ${\hat{\varphi }}^{\mathrm{tr}}$, is backpropagated to the spatial coordinates to obtain first-order differentials, the results are iteratively backpropagated to obtain higher-order differentials, i.e., the first and second-order spatial partial derivatives, $\nabla {\hat{\varphi }}^{\mathrm{tr}}=\left[\frac{\partial {\hat{\varphi }}^{\mathrm{tr}}}{\partial x},\frac{\partial {\hat{\varphi }}^{\mathrm{tr}}}{\partial y}\right]$, and $\nabla^{2}{\hat{\varphi }}^{\mathrm{tr}}=\frac{\partial^{2}{\hat{\varphi }}^{\mathrm{tr}}}{\partial x^{2}}+\frac{\partial^{2}{\hat{\varphi }}^{\mathrm{tr}}}{\partial y^{2}}$ are calculated by NN’s AD. The predicted spatial partial derivatives ($\nabla {\hat{\varphi }}^{\mathrm{tr}}$, $\nabla^{2}{\hat{\varphi }}^{\mathrm{tr}}$) are substituted to the formulation of the stab-EPT in Equation (3) to predict the conductivity, denoted as $\hat{\sigma }$. The predicted $\hat{\sigma }$ is compared with the reference padding conductivity in the boundary of the region of interest σ, i.e., the true ground values from the boundaries (boundary conditions), leading to another loss function, $L_{{\hat{\sigma }}_{\mathrm{BC}}}$,

(6) $$L_{{\hat{\sigma }}_{\mathrm{BC}}}=L_{MSE}\left(\sigma_{\mathrm{BC}}\;,{\hat{\sigma }}_{\mathrm{BC}}\right)+L_{SSIM}\left(\sigma_{\mathrm{BC}}\;,{\hat{\sigma }}_{\mathrm{BC}}\right)$$

As mentioned, Equation (3) has a diffusion term with a diffusion coefficient ρ. This coefficient is initially set according to bound values [1 × 10${}^{-6}$, 0.01] and a sigmoid activation function. The minimal value is a non-zero value to prevent the removal of the diffusion coefficient from the formulation. The maximum value is selected according to a grid search to find the global value for which the reconstruction is stable, i.e, without numerical artifacts [24]. The initial diffusion coefficient is then iterated by an incremental gradient-based optimization update according to the backpropagation of the weighted loss sum, $\lambda_{1}L_{\varphi^{\mathrm{tr}}}$ + $\lambda_{2}L_{{\hat{\sigma }}_{\mathrm{BC}}}$, as shown in Figure 1.

The NN is trained for 60,000 iterations in two steps. In the first step, the values of $\lambda_{1}$ and $\lambda_{2}$ are set to 100 and 0 during the first 10,000 iterations to prioritize the accuracy of $L_{\varphi^{\mathrm{tr}}}$. In the second step, $\lambda_{1}$ and $\lambda_{2}$ are set to 100 and 1, respectively, during the remaining iterations to balance both terms. The $\lambda_{1}$ and $\lambda_{2}$ values are selected empirically. The NN is composed of four fully-connected layers with 50 neurons per layer and an output layer with a single neuron. All layers are initialized by the default initialization parameter (Kaiming initialization) without bias terms [42]. All the neurons are activated with a sigmoid activation function. The NN output is constrained by the sigmoid activation function and scaled to the minimal and maximal values of the ground truth phase. The range of the NN hyper-parameters are selected according to literature [29,32,43], and the hyper-parameters were empirically selected within that range. The optimization algorithm used is Adam [44] with a learning rate of 0.001. The best model according to the loss function from the total number of iterations is used to test the accuracy of the reconstructions. For comparison purposes an additional method is created, where instead of the stab-EPT formulation in Equation (3), the std-EPT formulation (Equation (1)) is used to predict $\hat{\sigma }$ during the optimization. This method is referred to as PINN Std-EPT.

### 2.3. Numerical Simulations for Sample Preparations

**Figure 2:**
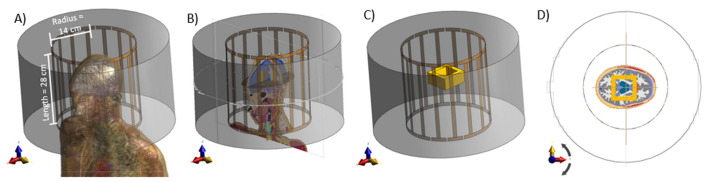
Numerical simulations for generating numerical samples from digital human phantoms. (**A**) a digital human phantom in a birdcage coil, (**B**) sectional views on the xy-, yz-, and xz-plane, (**C**) an extra reference padding tissue layer with known conductivity was added to the boundary of the region of interest (ROI), (**D**) the center slice of the digital human phantom.

Finite differences time domain (FDTD) electromagnetic software, Sim4Life${}^{©}$ (ZMT AG, Zurich), was used for simulations to prepare the samples. The RF coil was a shielded 16-rung birdcage coil as shown in Figure 2. The radius of the coil and the shield are 14 cm and 25 cm, respectively. The birdcage coil was excited in quadrature mode, with two ports 90${}^{\circ }$ apart geometrically, and a phase shift of 90${}^{\circ }$. The working frequency is 127.78 MHz, corresponding to 3 T of magnetic field strength.

For the reconstruction methods, the $B_{1}$ fields at the central slides were extracted. On these slices, the end-ring effects are less prominent, which suppresses the value of the change of the z-component of the *B* field ($B_{z}$), i.e., the change in the *B* field along the z-direction of the coil can be approximated to be zero ($\delta B_{z}\approx 0$). The coil was loaded with a digital human phantom, “Ella” from the virtual population 3.0 [45]. For the proposed PINN-MREPT the extra reference padding tissue layer with known conductivity added to the boundary of the ROI is shown in Figure 2D. The extracted part of the brain slice with the extra reference padding added to the boundary of the ROI is called **Ella phantom**. Although it looks impractical for the current ROI in Figure 2D, for clinical practice when the ROI is the whole head, this padding layer can be substituted for a helmet [46] with known conductivity. Moreover, circles with a diameter of 2 mm, 4 mm, and 6 mm and a dielectric conductivity of 1.2 [S/m] [47] were added to the Ella phantom to mimic cancerous tumors in the brain. This group of phantoms is called **Ella phantom with a tumor**. For each phantom, the B${}_{1}$-fields were calculated, and the corresponding σ and the phase of the B${}_{1}$-fields, $\varphi^{\mathrm{tr}}$ at the center of the coil, were extracted. The spatial resolution for all phantoms is 2 mm for the x-, y-, and z-direction. The region of interest for the reconstruction is a 2-D matrix of 30 × 30 pixels.

### 2.4. Noise

To test the noise robustness, zero-mean Gaussian distributed noise was added to the extracted $\varphi^{\mathrm{tr}}$ from the numerical simulations. The noise standard deviation is assumed to be $1/(\sqrt{2}\;\;\mathrm{SNR})$ according to [35] where SNR is the signal to noise ratio. The $\varphi^{\mathrm{tr}}$ contaminated with noise was used to test the accuracy of the reconstruction methods. The expected SNR values for an MREPT protocol are very dependent on the sequence and device, it has been mentioned in [35], that measurements with an approximate 50–100 SNR are an approximation in MREPT studies at 3 Tesla. Moreover, to test the compatibility of the proposed method with the filtering methods previously proposed in [35], a low-pass Gaussian filter with one standard deviation was applied to the $\varphi^{\mathrm{tr}}$ contaminated with noise.

### 2.5. Phantom Preparation and Scanning

A phantom was prepared and scanned in the Hubei Cancer Hospital with a 3T Skyra MRI scanner (Siemens, Erlangen, Germany) illuminated by a quadrature body coil. The phantom’s background was filled with a saline solution, a 2.5 g/L Sodium Chloride, and vials in the foreground were filled with a 10 g/L Sodium Chloride solution, with an estimated conductivity of 0.5 S/m and 2.0 S/m [48], respectively. A multi-gradient echo (m-GRE) sequence was used to recover the transceive phase [49,50]. The acquisition parameters were defined according to the description in [50], with a flip angle of 15 degrees, repetition time (TR) of 84 milliseconds, a first echo time (TE) of 1.91 milliseconds, a delta echo time of 2.67 milliseconds with 6 echoes in total. The resolution in the x-, y- directions are 1.06 mm, 1.06 mm, respectively, and a slice thickness of 2 mm. The phase maps at all TEs were fitted to the phase measurement at TE = 0 by temporal fitting [49,50], to recover the transceive phase. Between the background and foreground there is a 3 mm acrylic wall separation, this wall does not generate any MR phase signal, which produces artifacts in the conductivity reconstruction. The recovered transceive phase is filtered as mentioned in Section 2.4, to reduce the artifacts generated by the wall and noise in other areas. The peripheral outermost 3 pixels were taken as the reference padding with a known conductivity of 0.5 S/m.

## 3. Results

**Figure 3:**
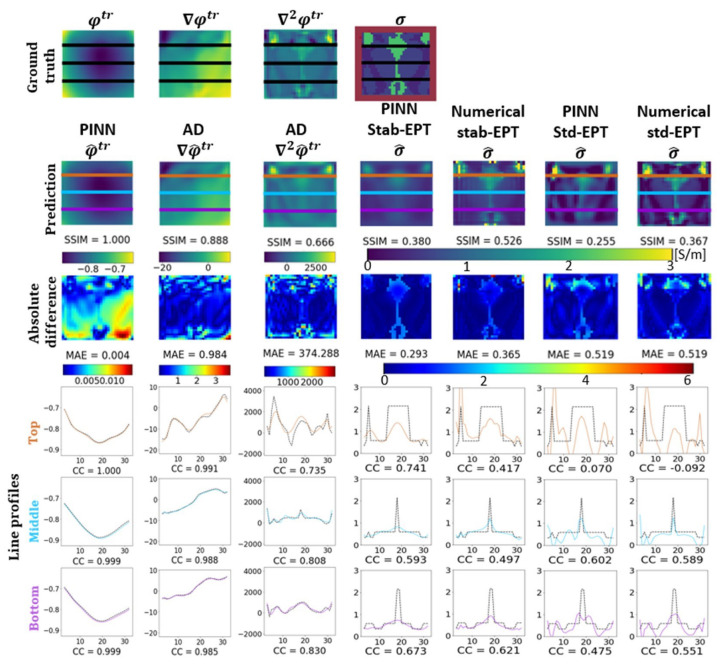
The ground-truth phase, the reconstructed phase and its differentiation ($\varphi^{\mathrm{tr}}$, $\nabla \varphi^{\mathrm{tr}}$, $\nabla^{2}\varphi^{\mathrm{tr}}$) and the reconstructed conductivity distribution of the noise-free **Ella phantom**. The items to compare from row 2 onward are PINN predicted phase, AD (columns 2–3, AD calculated gradient and Laplacian of phase, respectively), PINN Stab-EPT, numerical stab-EPT, PINN Std-EPT, and numerical std-EPT from left to right. From the top to down, row 1 shows the ground truth, row 2 shows the reconstructed maps with the corresponding SSIMs labeled, and row 3 shows the corresponding maps of the absolute difference between the reconstructed maps in row 2 and the ground truth in row 1. The last three rows show the values along the top (brown), middle (blue), and bottom (purple) horizontal lines of the maps in rows 1–2, respectively, with the corresponding correlation coefficients (CCs) labeled. Note that the phase, gradient, and Laplacian in the figure are the predictions from PINN Stab-EPT. For keeping the readability of the graphs, those of PINN Std-EPT are not shown in this graph. However, they can be found in Figure A5, Figure A6, Figure A7 and Figure A8 in Appendix A. This simplification is also applied to Figure 5 and Figure 6.

**Figure 4:**
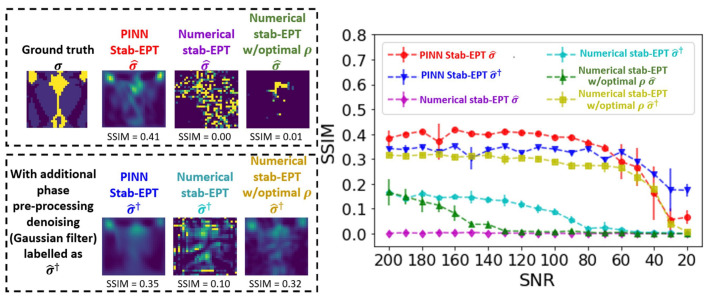
Noise robustness of various methods. (**Left**) Reconstructed conductivity of the **Ella phantom** at 200 SNR by PINN Stab-EPT, numerical stab-EPT, and using the numerical stab-EPT with the diffusion coefficient (ρ) optimized by PINN (legend ’Numerical stab-EPT w/optimal ρ$\hat{\sigma }$’) in the top row. In the second row, the same reconstruction methods are shown with the denoising pre-processing in Section 2.4 applied to $\varphi^{\mathrm{tr}}$, PINN Stab-EPT (’PINN Stab-EPT ${\hat{\sigma }}^{†}$’), numerical stab-EPT (’Numerical stab-EPT ${\hat{\sigma }}^{†}$’), and numerical stab-EPT with the ρ optimized by PINN (’Numerical stab-EPT w/optimal ρ${\hat{\sigma }}^{†}$’). (**Right**) Average and standard deviations of SSIM of the reconstructions of the conductivity for five trials from 200 to 20 SNR at a step of 10.

**Figure 5:**
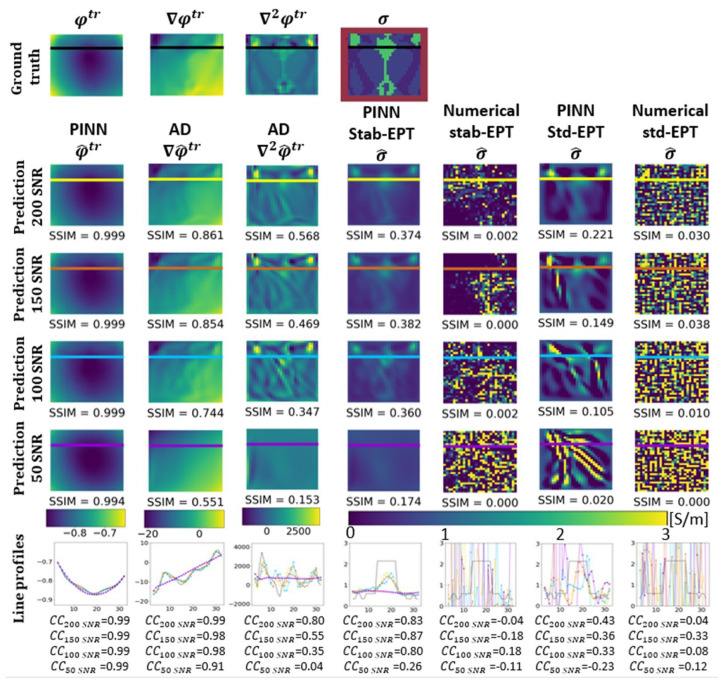
The ground-truth phase, the reconstructed phase, and its differentiation ($\varphi^{\mathrm{tr}}$, $\nabla \varphi^{\mathrm{tr}}$, $\nabla^{2}\varphi^{\mathrm{tr}}$) and the reconstructed conductivity distribution of the noise-contaminated **Ella phantom**. The items to compare from row 2 onward are PINN predicted phase, AD (columns 2–3, AD calculated gradient and Laplacian of phase, respectively), PINN Stab-EPT, numerical stab-EPT, PINN Std-EPT, and numerical std-EPT from left to right. From the top to down, row 1 shows the ground truth, and rows 2–5 show the reconstructed maps at different input SNRs with the corresponding SSIMs labeled. The last row shows the values along the colored horizontal lines indicated in the maps in rows 1–5 with the corresponding correlation coefficients (CCs) labeled.

**Figure 6:**
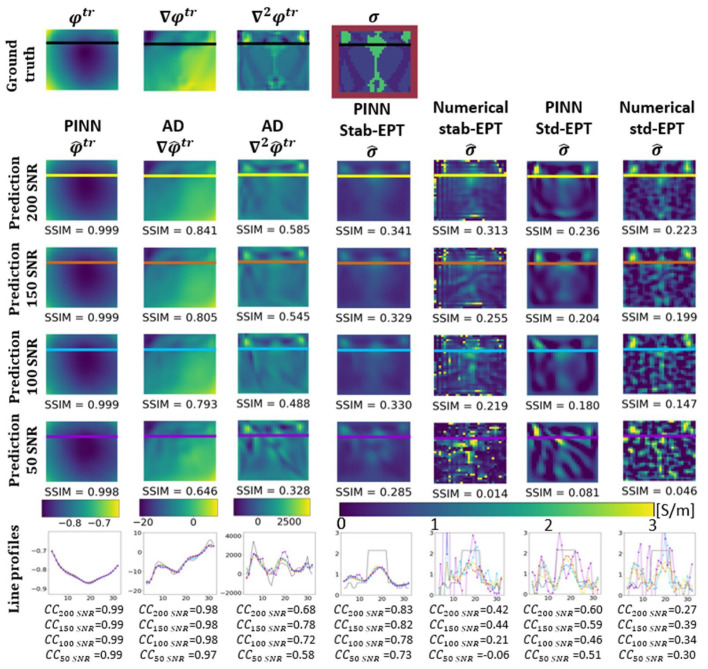
The ground-truth phase, the reconstructed phase, and its differentiation ($\varphi^{\mathrm{tr}}$, $\nabla \varphi^{\mathrm{tr}}$, $\nabla^{2}\varphi^{\mathrm{tr}}$) and the reconstructed conductivity distribution of the noise-contaminated **Ella phantom** with a pre-processing noise filter applied. The items to compare from row 2 onward are PINN predicted phase, AD (columns 2–3, AD calculated gradient and Laplacian of phase, respectively), PINN Stab-EPT, numerical stab-EPT, PINN Std-EPT, and numerical std-EPT from left to right. From the top to down, row 1 shows the ground truth, and rows 2–5 show the reconstructed maps at different input SNRs with the corresponding SSIMs labeled. The last row shows the values along the colored horizontal lines indicated in the maps in rows 1–5 with the corresponding correlation coefficients (CCs) labeled.

**Figure 7:**
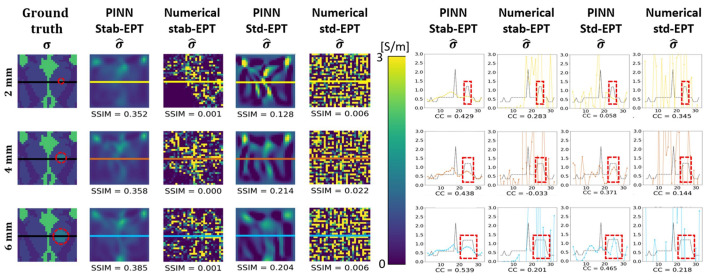
Ground truth and reconstructed conductivity of the **Ella phantom with a tumor** of different sizes. Column 1 is the ground truth conductivity. Columns 2–5 show the reconstructed conductivity maps using PINN Stab-EPT, numerical stab-EPT, PINN Std-EPT, and numerical std-EPT, respectively. From the top to down, rows 1–3 are maps with a mimicking circular cancerous region with a radius of 2, 4, and 6 mm, respectively. The red dashed circles in the first column indicate the location of the tumor. Columns 6–9 show the reconstructed values along the colored horizontal lines indicated in the maps in columns 1–5 with the corresponding CCs labeled. The red dashed boxes in Columns 6–9 show the location of the added tumors in the line profiles.

In this section, the results of several experiments are shown to validate our approach. Figure 3 shows the reconstructed phase and conductivity of the noise-free **Ella phantom**. The results were obtained by the two proposed PINN-MREPTs: PINN Stab-EPT and PINN Std-EPT and by their corresponding numerical methods: stab-EPT and std-EPT. The results are compared in terms of reconstruction accuracy (SSIM and mean absolute error (MAE)). The reconstructed values along the 3 specific horizontal lines (as shown in the second row of the figure) were further extracted, i.e., the line profiles, for further comparisons. The noise robustness of these MREPT methods was further examined. On the left of Figure 4, the reconstructions of **Ella phantom** at 200 SNR using different MREPT methods are shown. On the right of Figure 4, the reconstruction accuracy values when the input phase was set at 19 different SNR levels from 200 to 20 at a step of 10 are presented. Figure 5 shows the reconstructions of the **Ella phantom** with noise-contaminated phase information at four SNR levels (200, 150, 100, 50 SNR). Figure 6 shows the results when applying denoising as a pre-processing to the noise-contaminated phase information of the **Ella phantom**. For the **Ella phantom with a tumor**, the reconstructed results at different SNR levels are shown in Figure 7 and tabulated for each tissue type in Table 1.

### 3.1. Conductivity Reconstruction

**Figure A5:**
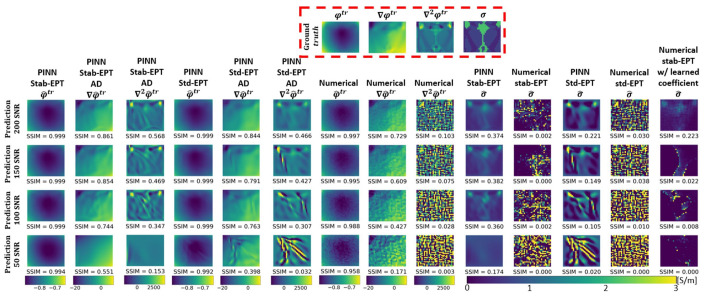
Reconstruction of **Ella phantom**. The ground-truth $\varphi^{\mathrm{tr}}$, $\nabla \varphi^{\mathrm{tr}}$, $\nabla^{2}\varphi^{\mathrm{tr}}$ and σ are shown on the dashed red box at the top. From row 2 onward, on columns 1–9, from left to right, it shows the phase and its differentiation (${\hat{\varphi }}^{\mathrm{tr}}$, the $\hat{\nabla }\varphi^{\mathrm{tr}}$, and ${\hat{\nabla }}^{2}\varphi^{\mathrm{tr}}$) predicted by PINN Stab-EPT, then the same categories predicted by PINN Std-EPT and the numerically calculated ones. After that, the predicted $\hat{\sigma }$ by PINN and numerical stab-EPT, followed by PINN and numerical std-EPT, and the numerical stab-EPT with optimal ρ are shown. From the top down, the predictions are made at 200, 150, 100, and 50 SNR, respectively. The SSIM values are indicated below each reconstruction.

**Figure A6:**
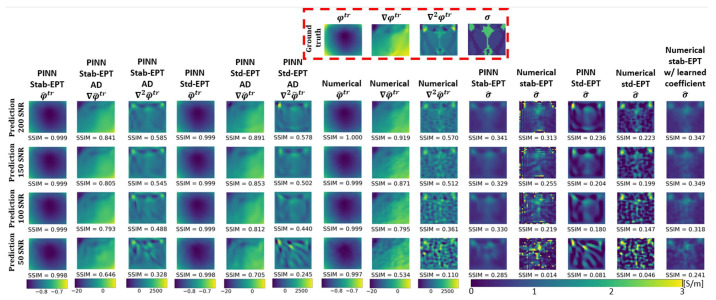
Reconstruction of **Ella phantom** with denoising pre-processing applied. The ground-truth $\varphi^{\mathrm{tr}}$, $\nabla \varphi^{\mathrm{tr}}$, $\nabla^{2}\varphi^{\mathrm{tr}}$ and σ are shown on the dashed red box at the top. From row 2 onward, on columns 1–9, from left to right, it shows the phase and its differentiation (${\hat{\varphi }}^{\mathrm{tr}}$, the $\hat{\nabla }\varphi^{\mathrm{tr}}$, and ${\hat{\nabla }}^{2}\varphi^{\mathrm{tr}}$) predicted by PINN Stab-EPT, then the same categories predicted by PINN Std-EPT and the numerically calculated ones. After that, the predicted $\hat{\sigma }$ by PINN and numerical stab-EPT, followed by PINN and numerical std-EPT, and the numerical stab-EPT with optimal ρ are shown. From the top down, the predictions are made at 200, 150, 100, and 50 SNR, respectively. The SSIM values are indicated below each reconstruction.

**Figure A7:**
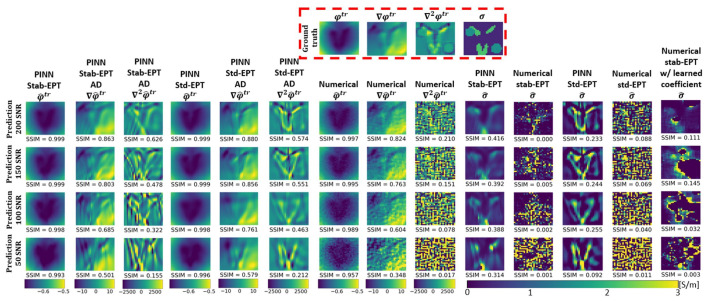
Reconstruction of **Duke phantom**. The ground-truth $\varphi^{\mathrm{tr}}$, $\nabla \varphi^{\mathrm{tr}}$, $\nabla^{2}\varphi^{\mathrm{tr}}$ and σ are shown on the dashed red box at the top. From row 2 onward, on columns 1–9, from left to right, it shows the phase and its differentiation (${\hat{\varphi }}^{\mathrm{tr}}$, the $\hat{\nabla }\varphi^{\mathrm{tr}}$, and ${\hat{\nabla }}^{2}\varphi^{\mathrm{tr}}$) predicted by PINN Stab-EPT, then the same categories predicted by PINN Std-EPT and the numerically calculated ones. After that, the predicted $\hat{\sigma }$ by PINN and numerical stab-EPT, followed by PINN and numerical std-EPT, and the numerical stab-EPT with optimal ρ are shown. From the top down, the predictions are made at 200, 150, 100, and 50 SNR, respectively. The SSIM values are indicated below each reconstruction.

**Figure A8:**
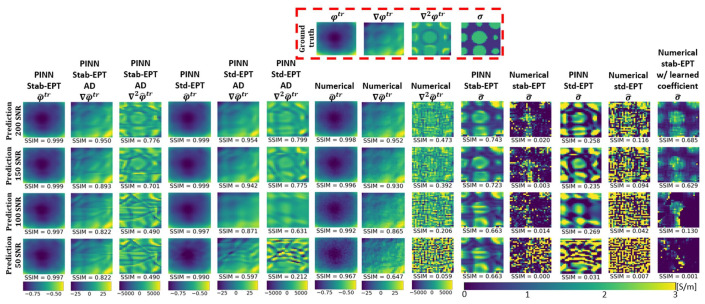
Reconstruction of **artificial geometry phantom**. The ground-truth $\varphi^{\mathrm{tr}}$, $\nabla \varphi^{\mathrm{tr}}$, $\nabla^{2}\varphi^{\mathrm{tr}}$ and σ are shown on the dashed red box at the top. From row 2 onward, on columns 1–9, from left to right, it shows the phase and its differentiation (${\hat{\varphi }}^{\mathrm{tr}}$, the $\hat{\nabla }\varphi^{\mathrm{tr}}$, and ${\hat{\nabla }}^{2}\varphi^{\mathrm{tr}}$) predicted by PINN Stab-EPT, then the same categories predicted by PINN Std-EPT and the numerically calculated ones. After that, the predicted $\hat{\sigma }$ by PINN and numerical stab-EPT, followed by PINN and numerical std-EPT, and the numerical stab-EPT with optimal ρ are shown. From the top down, the predictions are made at 200, 150, 100, and 50 SNR, respectively. The SSIM values are indicated below each reconstruction.

Figure 3 shows the results of the noise-free **Ella phantom**. The first row from left to right, shows the noise-free ground truth phase ($\varphi^{\mathrm{tr}}$), its spatial derivatives ($\nabla \varphi^{\mathrm{tr}}$, and $\nabla^{2}\varphi^{\mathrm{tr}}$) calculated by the Savistzky-Golay filter [51] with a second order polynomial, and the ground truth conductivity distribution (σ) of the **Ella phantom**, i.e., the digital human brain phantom “Ella” with an extra reference padding layer of three-pixel thick with conductivity of 1.0 S/m (highlighted in red). The second row from left to right, shows the prediction of the ${\hat{\varphi }}^{\mathrm{tr}}$ by PINN, $\nabla {\hat{\varphi }}^{\mathrm{tr}}$, $\nabla^{2}{\hat{\varphi }}^{\mathrm{tr}}$ by AD, the predicted $\hat{\sigma }$ by the proposed PINN Stab-EPT, the numerical stab-EPT with a fixed diffusion coefficient (ρ = 0.001) (found by a grid search to produce the best reconstruction in terms of SSIM), the PINN Std-EPT and the numerical std-EPT. The corresponding reconstruction accuracy in terms of SSIM is labeled below each reconstructed map. The third row shows the corresponding absolute difference maps between the reconstructed maps in row 2 and the ground truth in row 1, and the MAE values are indicated. The last three rows show plots of the values along the top (brown), middle (blue), and bottom (purple) horizontal lines of the maps in rows 1–2, respectively, and the corresponding correlation coefficients (CCs). Note that the phase, gradient, and Laplacian in the figure are the predictions from PINN Stab-EPT. For keeping the readability of the graphs, those of PINN Std-EPT are not shown in this graph. However, they can be found in Figure A5, Figure A6, Figure A7 and Figure A8 in the appendix of this manuscript in Appendix A. This simplification is also applied to Figure 5 and Figure 6.

As shown in Figure 3, the reconstructed conductivity by the numerical methods present the artifacts described in Section 2.1. PINN Std-EPT shows similar artifacts as the numerical std-EPT, while PINN Stab-EPT does not. The PINN Stab-EPT shows a smaller SSIM than numerical stab-EPT (0.380 vs. 0.526), but a better MAE (as shown in the third row) (0.293 vs. 0.365). The artifact makes the difference.

Moreover, comparing the line profiles of the reconstructions in columns 4–5, it can be seen that PINN Stab-EPT presents fewer overestimated conductivity values than the numerical stab-EPT, PINN Std-EPT, and numerical Std-EPT, which is well reflected by the higher CCs of the line profiles.

Furthermore, the predicted ${\hat{\varphi }}^{\mathrm{tr}}$ and $\nabla {\hat{\varphi }}^{\mathrm{tr}}$ are similar to the ground truth, which is reflected by the SSIM values (SSIM = 0.888). Comparatively, the SSIM of the predicted $\nabla^{2}{\hat{\varphi }}^{\mathrm{tr}}$ drops to 0.666, which impacts the accuracy of the reconstruction of the PINN Std-EPT directly, as Equation (1) is composed solely of the second order derivative, as shown by the comparison with numerical Std-EPT (SSIM: 0.255 vs. 0.367). In summary, in noise-free reconstruction, PINN Stab-EPT and PINN Std-EPT show worse performance than their numerical counterparts.

### 3.2. Noise Robustness

The noise robustness of the proposed PINN-MREPT reconstruction was compared in Figure 4 by the reconstructions of the **Ella phantom** with different noise levels. The methods to compare are PINN Stab-EPT, numerical stab-EPT, and the numerical stab-EPT with the diffusion coefficient (ρ) optimized by PINN (’Numerical stab-EPT w/ optimal ρ’), and the same methods with the application of filtering explained in Section 2.4 as a pre-processing (denoted as ’PINN Stab-EPT ${\hat{\sigma }}^{†}$’, ’Numerical stab-EPT ${\hat{\sigma }}^{†}$’, ’Numerical stab-EPT w/ optimal ρ${\hat{\sigma }}^{†}$’). Reconstructions at 200 SNR are shown for the **Ella phantom**, on the left of Figure 4. The corresponding SSIMs are labeled below each map. The mean and standard deviations of the SSIMs of the reconstructions of five trials at 19 different SNR levels from 200 to 20 with a step of 10 using the above-mentioned MREPT methods are plotted on the graph to the right of Figure 4.

It can be seen that the PINN Stab-EPT reconstruction accuracy is higher compared with that of any other methods up to 50 SNR. It is worth noting that the accuracy reduces when a significant amount of noise is introduced (SNR < 50). In those cases, the highest accuracy passes to the PINN Stab-EPT with denoising pre-processing. On the other hand, the numerical-based methods show an SSIM below 0.2, approximately half of that of the PINN methods, even though the denoising filter or the optimized coefficient was applied. Regardless, when both methods are combined the accuracy is seen to approximate that of the PINN Stab-EPT ${\hat{\sigma }}^{†}$. Moreover, in the reconstructions on the left of the graph, it can be seen that reconstructions of the PINN Stab-EPT ${\hat{\sigma }}^{†}$ and Numerical stab-EPT w/ optimal ρ${\hat{\sigma }}^{†}$ are similar, but not as good as that of the PINN Stab-EPT. Overall, for the SNR levels higher than 50 SNR, the small standard deviations of the data from the five trials for all the PINN reconstruction methods show a robust reconstruction. On the other hand, for the SNR levels below 50, due to the amount of noise, the reconstructions present more variations.

Figure 5 shows the reconstructions of the **Ella phantom** as well as the line profiles at various noise levels. In the first row, the ground truth $\varphi^{\mathrm{tr}}$, the noise-free $\nabla \varphi^{\mathrm{tr}}$, $\nabla^{2}\varphi^{\mathrm{tr}}$ and ground truth σ of the **Ella phantom** are shown. In the second to the fifth row, the reconstructions at 200, 150, 100, and 50 SNR are shown, respectively. The last row shows the line profiles for the reconstructions at every noise level with corresponding correlation coefficients (CCs). The columns, from left to right, show the ${\hat{\varphi }}^{\mathrm{tr}}$, $\nabla {\hat{\varphi }}^{\mathrm{tr}}$, $\nabla^{2}{\hat{\varphi }}^{\mathrm{tr}}$, and predicted $\hat{\sigma }$ by the PINN Stab-EPT, followed by the numerical stab-EPT, the PINN Std-EPT, and the numerical std-EPT. The reconstructed ${\hat{\varphi }}^{\mathrm{tr}}$ is accurate throughout SNR levels presented. However, the $\nabla {\hat{\varphi }}^{\mathrm{tr}}$, $\nabla^{2}\varphi^{\mathrm{tr}}$ at 50 SNR lose important information about the tissue structure of the brain slice. The line profiles also reflect the fact by showing a straight line for 50 SNR. The prediction of the spatial partial derivatives affects the PINN Stab-EPT reconstruction directly. At SNR levels higher than 50, the reconstructions were similar, however, as shown in the fifth row and the sixth row, as predicted partial derivatives turn flat at 50 SNR, the reconstructed conductivity and its line profile are over-smoothed. On the other hand, the numerical reconstructions are completely biased by the noise with SSIM values below 0.038 throughout all the SNR levels as compared with Figure 5.

Figure 6, shows the same structure as Figure 5. This figure presents the reconstruction results at 200, 150, 100, and 50 SNR with the denoising filter described in Section 2.4 applied to all reconstruction methods as a pre-processing. For the two PINN methods shown in the fourth and sixth column, the predicted ${\hat{\varphi }}^{\mathrm{tr}}$, $\nabla {\hat{\varphi }}^{\mathrm{tr}}$, and $\nabla^{2}{\hat{\varphi }}^{\mathrm{tr}}$ show a certain similarity for all SNR levels, which result in similar reconstruction accuracy. For the numerical methods, the denoising process improves the reconstruction accuracy. However, the reconstruction accuracy decreases with the SNR level, and a rough noise-like texture can be seen on the numerical std-EPT due to the noise. Moreover, in the numerical stab-EPT, numerical artifacts are present which reduce the accuracy of the reconstruction. Furthermore, comparing the PINN Stab-EPT reconstructions in Figure 6 to Figure 5, it can be seen that the latter shows an improvement over the former in accuracy for the 200, 150 and 100 SNR. Meanwhile, the former presents a higher SSIM at 50 SNR, where the reconstruction of the former loses important information about the tissue structure of the brain slice. Additionally, the line profiles at 50 SNR of the reconstructions of Figure 6 present higher CCs than those from Figure 5.

### 3.3. Tissue Sensitivity

The tissue sensitivity was investigated by embedding a cancer-like tissue to form the **Ella phantom with a tumor**. The cylindrical cancer-like structure of either 2, 4, or 6 mm radius was placed at the same site in the phantom. The digital samples with the embedded cancer-like tissues are shown in the first column of Figure 7, followed by the reconstructions by the PINN Stab-EPT, numerical Stab-EPT, PINN Std-EPT, and numerical std-EPT in the second to fifth columns. SSIM values are shown below each reconstruction. In columns 6–9, the corresponding line profiles for each reconstruction with the corresponding correlation coefficients (CCs) are shown. As shown, the numerical reconstructions on the third and fifth columns are severely distorted by noise with SSIMs that is lower than 0.1. The PINN reconstructions on the second and fourth columns are shown to have a maximal SSIM of 0.385 and 0.214 for PINN Stab-EPT and PINN Std-EPT, respectively, with an SSIM varied with the size of the tumor.

For the line profiles at the right in Figure 7, it is observed that the results of the PINN Stab-EPT in column 6 show the highest CCs among the four methods. Within the cancer-like area, PINN Std-EPT shows the highest accuracy although the corresponding CCs are not the highest, which is due to the over or under-estimations of $\hat{\sigma }$ outside the region.

Table 1 lists the reconstructed conductivity values for each tissue type in the region of interest for the **Ella phantom with a tumor** at 100 SNR. The tissues are divided into cerebrospinal fluid (CSF), white matter, gray matter, and embedded cancer-like tissue of three sizes for each reconstruction method. The numerical reconstructions, due to their weakness to noise contamination, show higher values of standard deviations. The mean tissue conductivity values by the PINN Stab-EPT are closer to the ground truth and present the smallest standard deviations. It is worth noting that for the 2 mm tumor, the reconstruction of PINN Stab-EPT presents an underestimation compared with the PINN Std-EPT. This can be related to the reconstructions shown in Figure 7, where due to the application of the diffusion coefficient (ρ) and the small size of the cancer-like tissue, the reconstruction does not keep its contrast to its surrounding tissues.

### 3.4. Optimization Details

**Figure 8:**
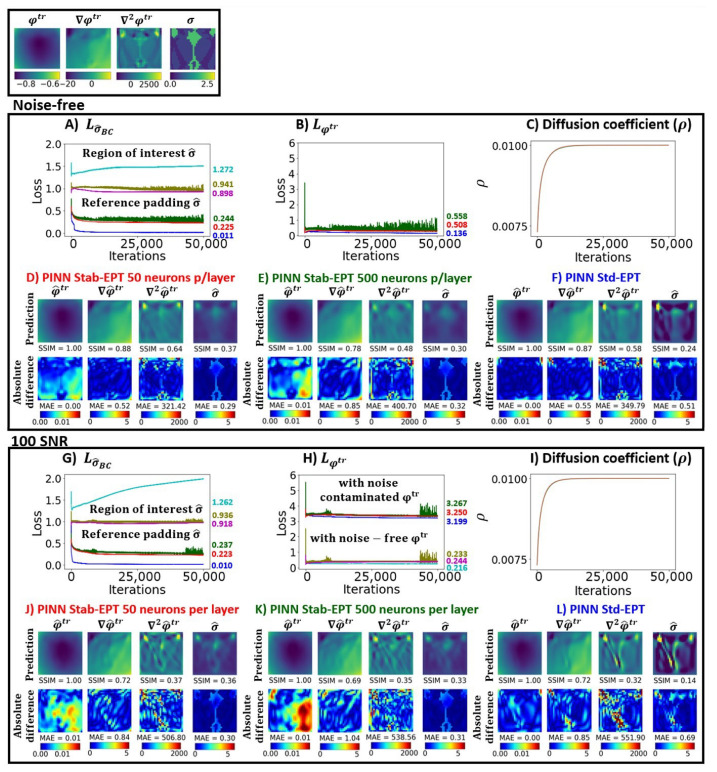
The ground truth (first box), loss curves, reconstructed conductivity, and phase of both the noise-free (second box) and noise-contaminated (100 SNR) (third box) of the **Ella phantom**. The loss curves of term $L_{{\hat{\sigma }}_{\mathrm{BC}}}$ and $L_{\varphi^{\mathrm{tr}}}$ are shown in (**A**,**B**,**G**,**H**). The diffusion coefficient value versus the iterations are shown in (**C**,**I**). The reconstructions and absolute difference maps of ${\hat{\varphi }}^{\mathrm{tr}}$, $\nabla {\hat{\varphi }}^{\mathrm{tr}}$, $\nabla^{2}{\hat{\varphi }}^{\mathrm{tr}}$, and $\hat{\sigma }$ are shown in the lower part of the second and third box for the noise-free and the noise-contaminated samples, respectively. The maps for PINN Stab-EPT (50 neurons per layer, labeled in red and called ’red case’), PINN Stab-EPT (500 neurons per layer, labeled in green and called ’green case’), and PINN Std-EPT (labeled in blue and called ’blue case’) are shown in (**D**) and (**J**), (**E**) and (**K**), (**F**) and (**L**), respectively. The SSIM and MAE values are indicated below each reconstruction or absolute difference map. In (**A**,**G**), two types of $L_{{\hat{\sigma }}_{\mathrm{BC}}}$ are shown. One is the loss regarding the conductivity in the surrounding extra reference padding (boundary conditions), depicted using red, green, and blue curves that correspond to the three different cases. The other type is the error regarding the region of interest, depicted using light red (magenta), light green (olive), and light blue (cyan) curves for the black, red, and blue cases, respectively. In (**B**,**H**), the red, green, and blue curves show the error between the predicted phase and the noise-contaminated ground truth. In (**H**) the light red, light green, and light blue curves show the error between the predicted phase and the noise-free ground truth. In (**A**,**B**,**G**,**H**), the minimum value of each loss and error curve is indicated on the right of its respective curve.

Figure 8 shows the ground truth (first box), the loss and error curves of the noise-free (second box) and noise-contaminated (100 SNR) (third box) **Ella phantom** to illustrate the optimization process. The loss curves in (A) and (G) show the $L_{{\hat{\sigma }}_{\mathrm{BC}}}$ loss term, the loss regarding the conductivity value in the reference padding. (B) and (H) show the loss curves regarding the $L_{\varphi^{\mathrm{tr}}}$ loss term, the loss regarding the phase value. The diffusion coefficient versus the iterations is shown in (C) for the noise-free sample and in (I) for the 100 SNR sample. The loss and error curves are shown for the PINN stab-EPT with the NN described in Section 2.2 (50 neurons per layer) in red and called ’red case’, PINN stab-EPT with a bigger NN (500 neurons per layer) in green and called ’green case’, and the PINN Std-EPT for a NN with 50 neurons per layer NN in blue and called ’blue case’. The error curve regarding the conductivity value in the region of interest is shown in lighter colors (red case in light red (magenta), green case in light green (olive), and blue case in light blue (cyan)) in (A) and (G). Additionally, (H) shows the error curve between the predicted phase and the noise-free ground truth phase in the same lighter color coding. The reconstructions and absolute difference maps of ${\hat{\varphi }}^{\mathrm{tr}}$, $\nabla {\hat{\varphi }}^{\mathrm{tr}}$, $\nabla^{2}{\hat{\varphi }}^{\mathrm{tr}}$, and $\hat{\sigma }$ are shown in the second box for the noise-free samples and in the third box for the noise-contaminated samples. The maps for PINN Stab-EPT’s red case (50 neurons per layer), green case (500 neurons per layer), and PINN Std-EPT’s blue case are shown in (D) and (J), (E) and (K), (F) and (L), respectively. The SSIM and MAE values are indicated below each reconstruction or absolute difference map.

As shown in (A) and (B), the bigger NN PINN Stab-EPT in green causes spikes in both reference-padding-conductivity and phase loss curves, compared with a smooth loss curve from the smaller NN PINN Stab-EPT in red. The same tendency can be observed in (G) and (H). The minimum value of the region-of-interest conductivity error does not show an apparent difference between the NNs with different size, as shown in (A) and (G).

Comparing PINN Stab-EPT (red loss curves) and PINN Std-EPT (blue loss curves) in (A), (B), (G), and (H), the latter showed the lowest reference padding conductivity loss and phase loss. Even the error of the noise-free phase shows the same tendency. However, PINN Stab-EPT resulted in lower region-of-interest conductivity errors in (A) and (G). The reconstructed conductivity distributions of the smaller NN PINN Stab-EPT, for the noise-free sample (in D)) and for the 100 SNR sample (in J)) show the highest SSIM and the lowest MAE. On the other hand, the region-of-interest conductivity errors of PINN Std-EPT in (A) and (G) turn higher after they reach their minimal value at around iteration 330.

Moreover, in (H), for both NNs, the error between the noise-free phase and predicted phase is lower than the loss regarding the noise-contaminated phase, 0.233, 0.244 vs. 3.267, 3.250, respectively, which shows that most of the noise does not appear in the reconstructed ${\hat{\varphi }}^{\mathrm{tr}}$. The same can be said for the PINN Std-EPT.

As seen from (C) and (I), the diffusion coefficient values converge (ρ=0.01) after approximately 13,000 iterations in both cases.

### 3.5. Phantom Reconstruction

**Figure 9:**
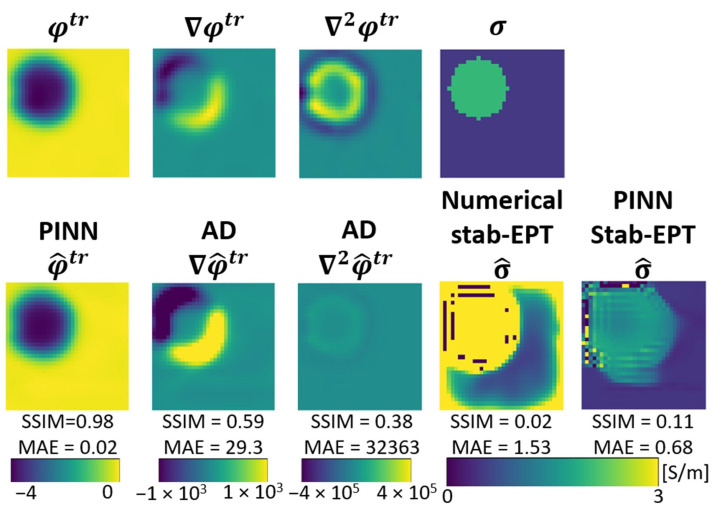
Phase and conductivity reconstruction for measurement of a phantom. In the first row, ground-truth phase, its numerical differentials ($\varphi^{\mathrm{tr}}$, $\nabla \varphi^{\mathrm{tr}}$, $\nabla^{2}\varphi^{\mathrm{tr}}$) and ground truth conductivity are shown. The PINN Stab-EPT predicted phase, AD calculated gradient and Laplacian of phase (columns 2–3, respectively), the reconstructed conductivity by numerical stab-EPT and PINN Stab-EPT are shown in the second row. The SSIM and MAE values are labelled below each reconstruction.

The reconstruction of the scanned phantom described in Section 2.5 is shown in Figure 9. In the first row from left to right, the ground truth conductivity, gradient, Laplacian, and conductivity are shown. In the second column the predicted phase by PINN and the Laplacian and gradient calculated by AD are shown, followed by the reconstructed conductivity by the numerical stab-EPT and by PINN Stab-EPT. The SSIM and MAE values are labeled below each reconstruction. The conductivity values above or below the expected realistic conductivity (0 S/m 5 S/m) are cut off. The numerical reconstruction contain noise, artifacts near the wall between the background and the foreground, and an over-estimation of the conductivity values. In comparison to the numerical stab-EPT, the PINN Stab-EPT could estimate both foreground and background conductivity, as shown by the MAE (1.53 vs. 0.68), however, it suffers from the influence of the noise and image boundary, too.

## 4. Discussion

### 4.1. Noise Robust Reconstructions

The noise sensitiveness is a major obstacle to the practical use of MREPT in clinical settings [2,3,4,52,53], where noise contamination is unavoidable. As shown in Figure 3, Figure 5 and Figure 6, the performance of the two analytic models deteriorate fast as the SNR decreases. The numerical error when calculating the spatial partial derivatives that are necessary for analytic EP reconstruction is one of the sources of artifacts. In this work, automatic differentiation (AD) by a phase prediction NN is the key to deal with the problem. The noise-filtering of the NN is discussed too. Moreover, the roles of a diffusion term introduced to the analytic models which are the base of the proposed PINN-MREPT were investigated.

#### 4.1.1. AD & Noise-Filtering of NN

The std-EPT formulation relies on the Laplacian of the phase ($\nabla^{2}\varphi^{\mathrm{tr}}$), which, by numerical calculation, is extremely noise sensitive. The noise effect of the std-EPT formulation has been thoroughly examined in [54]. As seen in Figure 6, as SNR decreases from 200 to 50, the SSIM of PINN Std-EPT reconstruction (column six) decreases from 0.236 to 0.081 and the features of the map are captured till an SNR of 100, while that of the numerical std-EPT (column seven) decreases from 0.223 to 0.046 and all reconstructions show a rough noise-like texture, more noticeable as the noise increases. The difference lies partly in that the Laplacian is calculated either by AD or numerical computation. Since, the gradient and Laplacian can be rapidly calculated by the AD of the phase prediction NN, which is optimized with the loss functions ($L_{\varphi^{\mathrm{tr}}}$, $L_{{\hat{\sigma }}_{\mathrm{BC}}}$), the errors from numerical computation can be compensated. However, the NN solves the partial differential equation for every measurements, i.e., for every measurement, the NN has to be trained. Therefore, the training from one sample cannot be used for other samples.

Another important factor for noise-robustness is the NN’s spectral bias [34], i.e., the NN of PINN focuses on low-frequency features while filtering out high-frequency features, which contain part of the noise. This noise-filtering effect could be recognized by the comparison in Figure 5. Compared with ground truth line profiles, the line profiles by the numerical std-EPT (Row 6 of Column 7) show more and higher ripples than that of the PINN Std-EPT (Row 6 of Column 6). The details behind the line profiles can be seen in Figure A5 of the Appendix.

As another analytic MREPT method, stab-EPT relies on the gradient and the Laplacian of the phase ($\nabla \varphi^{\mathrm{tr}}$ and $\nabla^{2}\varphi^{\mathrm{tr}}$). $\nabla^{2}\varphi^{\mathrm{tr}}$ is the term with the higher impact on the reconstruction [35]. Thus, from Figure 3 and Figure 5, a comparison between PINN Stab-EPT (column 4 of both figures) and numerical stab-EPT (column 5 of both figures), observations similar to those from the comparison of PINN Std-EPT and numerical std-EPT could be made.

When denoising pre-processing is applied, as seen in Figure 6, the reconstructions of both numerical stab-EPT (column 5), numerical std-EPT (column 7), and PINN Std-EPT (column 6) are improved over their corresponding reconstructions without denoising in Figure 5 (at SNR 200, SSIM 0.313, 0.223, 0.236 over 0.002, 0.030, 0.221). The denoising works effectively for numerical analytic models, though the improvement for the PINN Std-EPT is very limited. This could be understood by comparing the Laplacian by automatic differentiation in Figure 5 (without denoising) and Figure 6 (with denoising).

Furthermore, the denoising applied to the PINN Stab-EPT reconstruction decreases the SSIM from 0.374, 0.382, 0.360 at 200, 150, and 100 SNR, respectively, (Figure 5, Column 4) to 0.341, 0.329, 0.330 (Figure 6, Column 4). Figure 4 shows that the reconstruction performance of the PINN Stab-EPT keeps being the highest until 50 SNR. The fact that the denoising could improve the reconstruction of PINN Std-EPT, but contrarily, lower the performance of PINN Stab-EPT, revealed that there is room to further improve the PINN Std-EPT.

Additionally, when increasing the number of NN’s middle neurons, the error curves regarding both conductivity and reconstructed phase vibrate more than those of the small number of middle neurons, as shown in Figure 8, which means that the small NN is better for generalized AD of the phase reconstruction. This has been studied in [29,43,55]. Moreover, the fact that the vibrations presented in the error curves of both reconstructions for the samples without noise and with noise (100 SNR) in Figure 8, suggests that the vibration was caused by the learning of the increased number of middle neurons, rather than noise. It is worth noting that the noise model used represents only a portion of the noise found in clinical measurements, robustness to noise in the clinical environment field needs to be researched in the future. Regardless, the reconstruction of the phantom shown in Figure 9 shows the potential for clinical application.

#### 4.1.2. The Role and Optimization of the Diffusion Coefficient

The stab-EPT formulation is able to reduce the effect of noise due to the diffusion term $\nabla^{2}\gamma$, which acts as a viscosity regularization term on the reconstruction [25], i.e., the vibrations on the reconstructions are reduced. Moreover, the diffusion coefficient (ρ), regulates the effect of the diffusion term. However, this coefficient is usually selected empirically [16,25,37] which becomes a confounding factor in the reconstructions. To the best of our knowledge, the optimization of the diffusion coefficient automatically has only been explored in [24], up to a local (pixel-wise) diffusion coefficient optimization to improve the accuracy of the reconstructions. However, the optimization of the diffusion coefficient is made according to samples from a dataset, i.e., it is a multi-sample approach that requires the curation of an appropriate dataset.

Comparatively, in the proposed PINN Stab-EPT, instead of empirical selection or optimization based on a dataset, which are obstacles to its application in a clinical setting, the coefficient is single sample optimized automatically with the loss functions ($L_{\varphi^{\mathrm{tr}}}$ + $L_{{\hat{\sigma }}_{\mathrm{BC}}}$) as seen in Figure 8C,I. When the diffusion term was applied to the PINN framework, it helps to smooth both tissue boundary and noise, which could be reflected by the line profiles shown in column 4 of Figure 5. The correlation coefficient values of them are much higher than those from the line profiles by PINN Std-EPT and two numerical analytic models when SNR levels are higher than 50 SNR.

By comparing the effect of the diffusion term (SSIM of PINN Stab-EPT vs. SSIM of PINN Std-EPT in Figure 5, for the 4 SNR levels: 0.374, 0.382, 0.360, 0.174 vs. 0.221, 0.149, 0.105, 0.020), with that of denoising pre-processing (SSIM of PINN Std-EPT in Figure 6 vs. SSIM of PINN Std-EPT in Figure 5: 0.236, 0.204, 0.180, 0.081 vs. 0.221, 0.149, 0.105, 0.020), it is clear that the diffusion could play a bigger role in noise resistance than denoising pre-processing. This additional denoising pre-processing could only improve the PINN Stab-EPT at SNR levels lower than 50 SNR, as shown by the comparison between the SSIM of PINN Stab-EPT without denoising (0.374, 0.382, 0.360, 0.174 in Figure 5) and that of PINN Stab-EPT with denoising pre-processing (0.341, 0.329,0.330, 0.285 in Figure 6). For the samples with higher SNR levels, PINN Stab-EPT cannot benefit from but gets degraded by the denoising pre-processing.

Moreover, the phase prediction of the NN is also affected by the diffusion term. As shown in Figure A5 of the Appendix, the Laplacian of predicted phase of PINN Stab-EPT (Column 3) has higher SSIM than that of PINN Std-EPT for all the 4 SNR levels (0.568, 0.469, 0.347, 0.153 vs. 0.466, 0.427, 0.307, 0.032). That is, both the conductivity reconstruction and phase prediction benefit from the diffusion term.

Additionally, the fact that the PINN Std-EPT’s lower phase loss and reference padding conductivity loss do not lead to a lower region of interest conductivity error, shown in Figure 8A,G, indicates that the NN might be over-fitting in PINN Std-EPT to a local minimum. On the other hand, the fact that PINN Stab-EPT does lead to a decrease of loss of reference-padding conductivity, error of ROI conductivity, and loss of phase, suggests that (1) the diffusion term plays a role in balancing the phase and conductivity prediction, and (2) the loss containing diffusion term could guide the propagation of conductivity information of the reference padding to the region of interest. As mentioned above, the diffusion coefficient’s viscosity effect in Equation (4) dampens the variations in conductivity [25], i.e., it tightens the bonds among the conductivity values in the neighborhood of the pixel location where the diffusion value is applied [24]. The proposed PINN stab-EPT’s diffusion coefficient applies the diffusion term equally for both the reference padding and the region of interest conductivity. The tightening of the bonds between the reference padding and the region of interest conductivity elucidates the relation in the propagation of information from the reference padding to the region of interest conductivity in PINN stab-EPT. However, further investigation is necessary into the relationship between the error of the region of interest conductivity and the terms of the loss function.

#### 4.1.3. Tissue Sensitivity

As mentioned above, the diffusion term of PINN Stab-EPT reduces the contrast of the reconstruction. The conductivity values are averaged in the reconstruction in Table 1, while this effect is alleviated in the PINN Std-EPT, especially for low-conductivity tissues, such as white matter and gray matter, their higher standard deviations make the distinction of cancer-like tissues difficult. The standard deviations of the two numerical analytic models are much higher than those of the PINN Std-EPT. From the line profiles shown in Figure 7, it is difficult for the two numerical analytic methods to identify any sizes of cancer-like tissues, but PINN Stab-EPT and PINN Std-EPT can discern the cancer-like tissues above 4 mm from the others. However, the size of cancer-like tissue identified by the line profiles of PINN Std-EPT is likely to be much smaller than its ground-truth size. Therefore, it is very important to make it possible the detection of cancer-like tissues smaller than 4 mm and tissue contrast identical to the original EP distribution, which will be the aim of our future work.

### 4.2. Providing Collocation Points for Single Sample MREPT

In this work, an extra reference padding was added to the object to be scanned. The data points in the padding then were used to provide collocations points to guide the optimization of the PINN for MREPT. This idea was inspired by [46], where dielectric padding was used to modify the *B* field that was further employed as a constraint in the reconstruction, which is different from the idea and approach of this work. During the iterative process of PINN Stab-EPT, the PINN learns from the additive collocation points in the boundary area and gradually generalizes to the rest of the ROI, which is proved by the phase predictions and conductivity reconstruction in Figure 3, Figure 5 and Figure 6. In this way, a single sample MREPT, which is especially important for clinical use can be realized. Moreover, this setup is practical for the clinical implementation, e.g., by the helmet structure mentioned in Section 2.3 in the case of EP reconstruction for the head. As the next step of the PINN-MREPT study, we will create a reference conductivity helmet, serving as reference padding, and scan physical head phantoms with the padding.

### 4.3. Computational Cost

The solution of the stab-EPT formulation for an input image with a size of 30 × 30 pixels, including the numerical calculation of the spatial derivatives, takes approximately 1 s in a CPU (Intel^®^ Core^™^ i7-9750H at 2.6 GHz). Meanwhile, the std-EPT solution on the same system takes approximately 0.75 s. By taking advantage of the graphics processing unit (GPU), the 60,000 iterations proposed for one sample PINN Stab-EPT reconstruction takes approximately 40 min on an Nvidia GeForce RTX 2070 GPU for the 50 neurons per layer NN, and each iteration takes approximately 0.045 s. In comparison, the PINN Std-EPT, which ran on the same system, takes approximately 0.025 s per iteration, while, the 500 neurons per layer NN takes approximately 0.070 s per iteration. The calculation of the spatial partial derivatives by AD is made faster than the numerical calculation. Moreover, PINN Stab-EPT is slower than PINN Std-EPT, this difference can be attributed to the creation of the discretized PDE system of equations, their solution, and backpropagation in Equation (4) which takes approximately 0.03 s per iteration. Meanwhile, due to the simple formulation of PINN Std-EPT Equation (1), there is no need to produce a system of equations that reduces the computational time to 0.01 s per iteration. In this work, due to the computational cost constraints associated with the solution of the stab-EPT formulation, the samples size of the phase image is limited to a 30 × 30 pixels area. In a clinical measurement, the reconstruction area might be 256 × 256 pixels or higher depending on the scanner. In those cases, the reconstruction can be parallelized in various region of interest where the probable tumorous tissues might be located. Future work will focus on the implementation of more efficient solutions to the discretized stab-EPT formulation to reduce the total computational cost of the reconstruction.

## 5. Conclusions

In this work, we proposed a noise-robust PINN-based-MREPT reconstruction method based on PINN and an optimizable stab-EPT formulation. The results of numerical experiments show that the reconstruction of conductivity is robust up to 50 SNR and outperforms several reconstruction methods in direct comparison. Moreover, it is shown that the reconstructions of cancer-like tissues up to 4 mm at 100 SNR can be successfully retrieved, in contrast to the failure of other methods. This work is an important step in the reconstruction of the EPs in cases where numerical formulations fail due to low SNR. The reconstruction of a phantom measurement shows the advantage of PINN-MREPT. Nonetheless, experiments with more physical phantoms, and human subjects are necessary to validate the method for the clinical use. Future work will focus on techniques to reduce computational cost and to add the optimization of local diffusion coefficients to the formulation to further increase the accuracy of the reconstructions. 

## Figures and Tables

**Table 1:** Reconstructed tissue conductivity values of the **Ella phantom with a tumor** of different sizes at 100 SNR. Mean and standard deviations per tissue are indicated.

Tissue	Ground Truth	PINN stab-EPT	Numerical stab-EPT	PINN std-EPT	Numerical std-EPT
CSF	2.14 ± 0.00	1.04 ± 0.35	0.96 ± 11.1	1.00 ± 0.87	1.04 ± 4.08
White matter	0.34 ± 0.00	0.45 ± 0.12	−4.67 ± 146	0.34 ± 0.63	0.32 ± 4.05
Gray matter	0.58 ± 0.00	0.60 ± 0.19	6.30 ± 245	0.48 ± 0.67	0.39 ± 4.07
Tumor 2 mm	1.20 ± 0.00	0.66 ± 0.01	0.91 ± 6.80	0.64 ± 0.09	1.63 ± 4.15
Tumor 4 mm	1.20 ± 0.00	0.73 ± 0.04	1.04 ± 3.37	0.82 ± 0.12	1.63 ± 4.90
Tumor 6 mm	1.20 ± 0.00	0.77 ± 0.06	−0.60 ± 14.50	0.71 ± 0.44	0.46 ± 4.26

**Figure A1:**
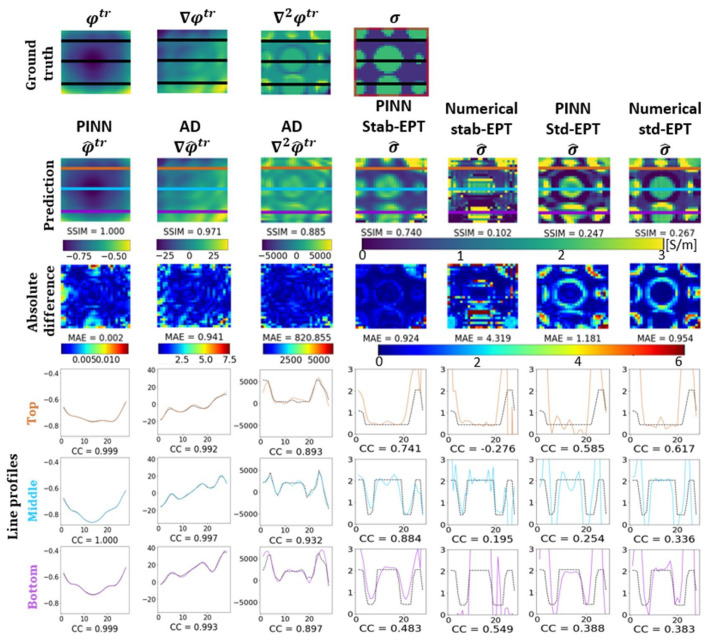
The ground-truth phase, the reconstructed phase and its differentiation ($\varphi^{\mathrm{tr}}$, $\nabla \varphi^{\mathrm{tr}}$, $\nabla^{2}\varphi^{\mathrm{tr}}$) and the reconstructed conductivity distribution of the noise-free **artificial geometry phantom**. The items to compare from row 2 onward are PINN predicted phase, AD (columns 2–3, AD calculated gradient and Laplacian of phase, respectively), PINN Stab-EPT, numerical stab-EPT, PINN Std-EPT, and numerical std-EPT from left to right. From the top to down, row 1 shows the ground truth, row 2 shows the reconstructed maps with the corresponding SSIMs labeled, and row 3 shows the corresponding maps of the absolute difference between the reconstructed maps in row 2 and the ground truth in row 1. The last three rows show the values along the top (brown), middle (blue), and bottom (purple) horizontal lines of the maps in rows 1–2, respectively, with the corresponding correlation coefficients (CCs) labeled.

**Figure A2:**
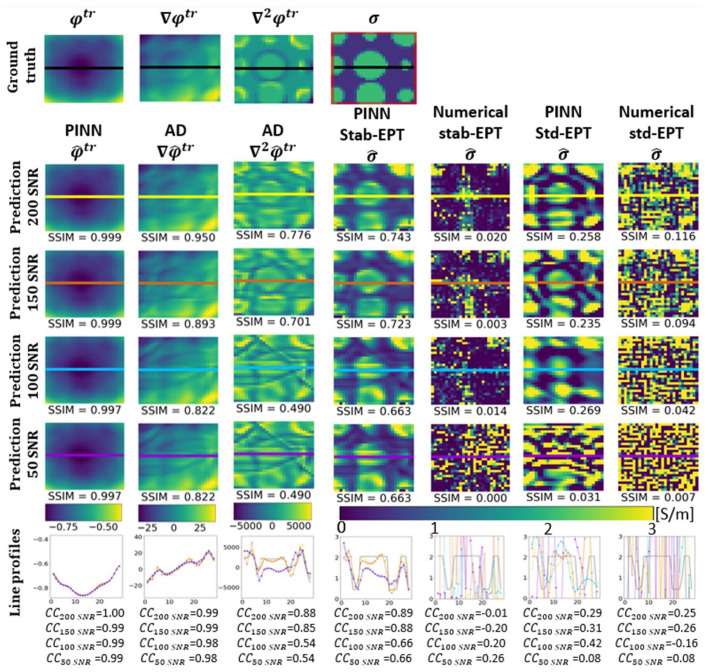
The ground-truth phase, the reconstructed phase, and its differentiation ($\varphi^{\mathrm{tr}}$, $\nabla \varphi^{\mathrm{tr}}$, $\nabla^{2}\varphi^{\mathrm{tr}}$) and the reconstructed conductivity distribution of the noise-contaminated of a slice of an **artificial geometry phantom**. The items to compare from row 2 onward are PINN predicted phase, AD (columns 2–3, AD calculated gradient and Laplacian of phase, respectively), PINN Stab-EPT, numerical stab-EPT, PINN Std-EPT, and numerical std-EPT from left to right. From the top to down, row 1 shows the ground truth, and rows 2–5 show the reconstructed maps at different input SNRs with the corresponding SSIMs labeled. The last row shows the values along the colored horizontal lines indicated in the maps in rows 1–5 with the corresponding correlation coefficients (CCs) labeled.

**Figure A3:**
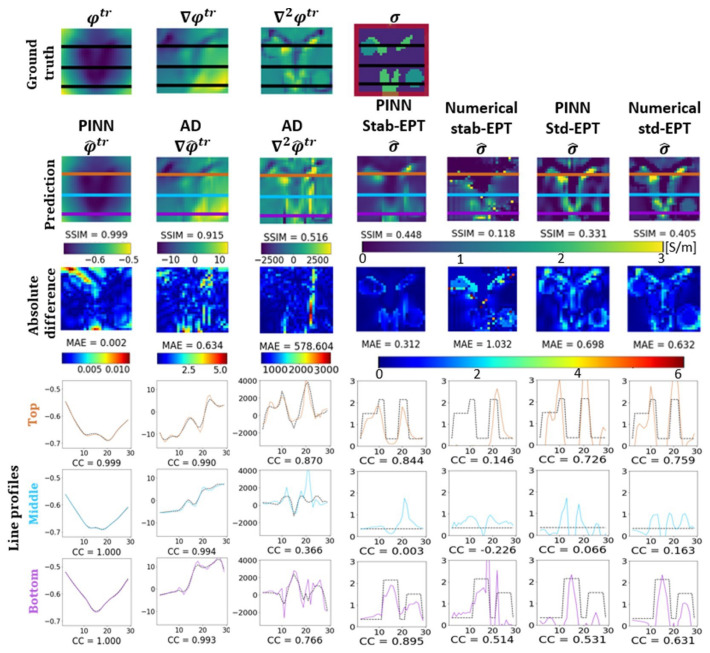
The ground-truth phase, the reconstructed phase and its differentiation ($\varphi^{\mathrm{tr}}$, $\nabla \varphi^{\mathrm{tr}}$, $\nabla^{2}\varphi^{\mathrm{tr}}$) and the reconstructed conductivity distribution of the noise-free **Duke phantom**. The items to compare from row 2 onward are PINN predicted phase, AD (columns 2–3, AD calculated gradient and Laplacian of phase, respectively), PINN Stab-EPT, numerical stab-EPT, PINN Std-EPT, and numerical std-EPT from left to right. From the top to down, row 1 shows the ground truth, row 2 shows the reconstructed maps with the corresponding SSIMs labeled, and row 3 shows the corresponding maps of the absolute difference between the reconstructed maps in row 2 and the ground truth in row 1. The last three rows show the values along the top (brown), middle (blue), and bottom (purple) horizontal lines of the maps in rows 1–2, respectively, with the corresponding correlation coefficients (CCs) labeled.

**Figure A4:**
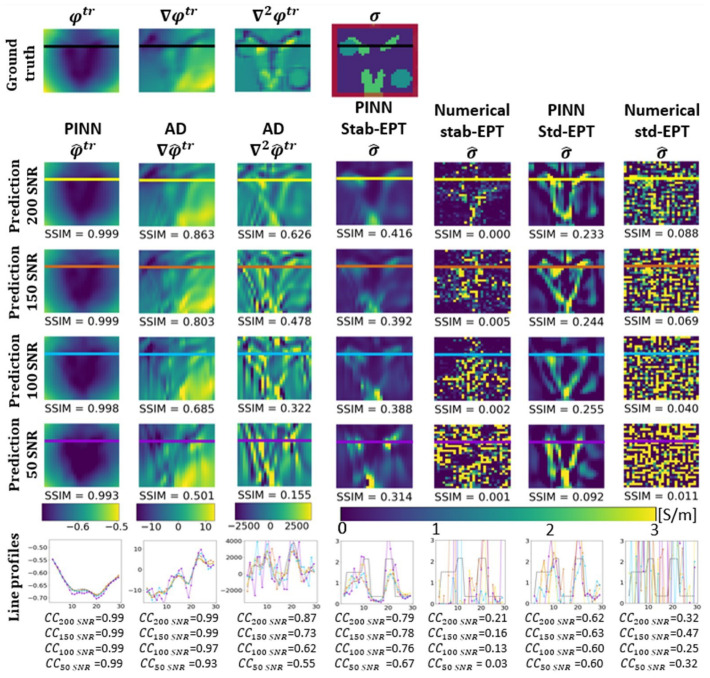
The ground-truth phase, the reconstructed phase, and its differentiation ($\varphi^{\mathrm{tr}}$, $\nabla \varphi^{\mathrm{tr}}$, $\nabla^{2}\varphi^{\mathrm{tr}}$) and the reconstructed conductivity distribution of the noise-contaminated **Duke phantom**. The items to compare from row 2 onward are PINN predicted phase, AD (columns 2–3, AD calculated gradient and Laplacian of phase, respectively), PINN Stab-EPT, numerical stab-EPT, PINN Std-EPT, and numerical std-EPT from left to right. From the top to down, row 1 shows the ground truth, and rows 2–5 show the reconstructed maps at different input SNRs with the corresponding SSIMs labeled. The last row shows the values along the colored horizontal lines indicated in the maps in rows 1–5 with the corresponding correlation coefficients (CCs) labeled.

**Figure A9:**
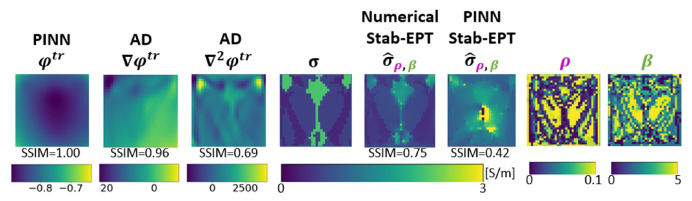
Reconstruction of conductivity distribution with known local coefficients (ρ,β) of the noise-free **Ella phantom**. The PINN Stab-EPT prediction of ${\hat{\varphi }}^{\mathrm{tr}}$, $\nabla {\hat{\varphi }}^{\mathrm{tr}}$, and $\nabla^{2}{\hat{\varphi }}^{\mathrm{tr}}$ are shown. The ground truth σ, numerical stab-EPT, and PINN Stab-EPT reconstruction $\hat{\sigma }$ with the optimized local coefficients (ρ, β) are shown to the right. The SSIM values of the reconstruction are shown below each reconstruction. Lastly, to the right, the applied ρ, and β are shown.

## Data Availability

The data supporting the conclusions of this article are available on request to the corresponding author.

## Appendix A

Two additional phantoms are produced and presented in the same manner as the **Ella phantom** to further test the reconstruction capabilities of the proposed method.

The reconstruction for a noise-free **artificial geometry phantom** results are shown in Figure A1. The phantom is composed of various circular structures with foregrounds of higher conductivity (2 [S/m]) compared with the background (0.5 [S/m]). The reference padding for the boundary conditions is only one pixel in width for each side in the boundary of the ROI. The figure is presented in the same manner as Figure 3. The numerical std-EPT and PINN Std-EPT-based reconstructions on the sixth and seventh columns are seen to produce artifacts near conductivity transitions. Numerical stab-EPT reconstructions with a fixed diffusion coefficient (ρ = 0.001) on the fifth column show many numerical artifacts. PINN Stab-EPT reconstructions on the fourth column show the main foregrounds of the phantom without additional artifacts. Moreover, the predictions of the ${\hat{\varphi }}^{\mathrm{tr}}$ and $\nabla {\hat{\varphi }}^{\mathrm{tr}}$ by PINN show small values of MAE and high values of SSIM, while for $\nabla^{2}{\hat{\varphi }}^{\mathrm{tr}}$ the error is increased, as seen on the line profiles on the last rows, the similarity is high as shown by the correlation coefficients (CC > 0.89).

Figure A2, shows the same structure as Figure 5 for the **artificial geometry phantom**. The ${\hat{\varphi }}^{\mathrm{tr}}$, $\nabla {\hat{\varphi }}^{\mathrm{tr}}$, and $\nabla^{2}{\hat{\varphi }}^{\mathrm{tr}}$ reconstructions by PINN shows a similar accuracy for all noise levels. The numerical reconstructions on the fifth and seventh columns are completely biased by the noise. The PINN Std-EPT reconstruction presents a similar structure to the ground truth from visual inspection, however, due to over and under-estimation of the conductivity, it presents SSIM values below 0.27, and the corresponding CCs also show big vibrations on the predicted $\hat{\sigma }$. Meanwhile, the PINN Stab-EPT formulation reconstructions on the fourth column maintain accuracy of SSIM > 0.663, through all noise levels and equally high accurate line profiles as the CCs are higher than 0.66.

Figure A3 shows the reconstruction of a digital human brain phantom “Duke” [45] with additional cancer-like tissues embedded referred as **Duke phantom** without noise. The reference padding for the boundary conditions is two pixels in width for each side in the boundary of the ROI. The structure of Figure A3 is the same as the one from Figure 3. It is seen, that the reconstruction shows an accuracy of 0.448 SSIM by the proposed PINN Stab-EPT, whereas the other reconstruction methods show over and under estimations of the conductivity as shown by the lower SSIMs 0.118, 0.331, 0.405 for the numerical stab-EPT, PINN Std-EPT, and numerical std-EPT, respectively. The corresponding noise-contaminated phase reconstructions are shown in Figure A4, at 200, 150, 100, and 50 SNR. Figure A4, shows the same structure as Figure 5 for the **Duke phantom**. It is shown that the PINN Stab-EPT reconstruction is robust for all noise levels (SSIM > 0.31). Meanwhile, the numerical stab-EPT, PINN Std-EPT, and std-EPT show minimal SSIM values of 0.00, 0.092, and 0.011, respectively.

For comparison purposes, in Figure A5, it shows the reconstructions for the **Ella phantom** at 200 SNR, 150 SNR, 100 SNR, and 50 SNR. The ground truth $\varphi^{\mathrm{tr}}$, and subsequent $\nabla \varphi^{\mathrm{tr}}$, and $\nabla^{2}\varphi^{\mathrm{tr}}$ numerically calculated by a second-order polynomial by the Savistzky-Golay filter and σ are shown on the dashed red box in the first row. Next, from left to right, the PINN Stab-EPT predicted ${\hat{\varphi }}^{\mathrm{tr}}$, and AD calculated $\nabla {\hat{\varphi }}^{\mathrm{tr}}$, and $\nabla^{2}{\hat{\varphi }}^{\mathrm{tr}}$ are shown on the first three columns. Following on the next three columns, the PINN Std-EPT predictions of ${\hat{\varphi }}^{\mathrm{tr}}$, and AD calculated $\nabla {\hat{\varphi }}^{\mathrm{tr}}$, and $\nabla^{2}{\hat{\varphi }}^{\mathrm{tr}}$ are shown. To the right, the noise-contaminated $\varphi^{\mathrm{tr}}$ and the numerically calculated $\nabla \varphi^{\mathrm{tr}}$, and $\nabla^{2}\varphi^{\mathrm{tr}}$ are shown. Moreover, the reconstruction of $\hat{\sigma }$ by PINN Stab-EPT, numerical stab-EPT, PINN Std-EPT, numerical std-EPT and numerical stab-EPT with the optimized diffusion coefficients learned from PINN Stab-EPT are shown on the tenth to the fourteenth columns, respectively. Figure A6 shows the same structure as Figure A5, for the **Ella phantom** with the pre-process filter described in Section 2.4. Meanwhile, Figure A7 and Figure A8 shows the same structure as Figure A5, for the **Duke phantom** and for the **artificial geometry phantom**, respectively.

Lastly, in Figure A9, optimized local diffusion and convection coefficients based on [24] are applied to the PINN Stab-EPT optimization. However, since the optimization is based on numerical spatial partial derivatives, and the prediction of $\nabla {\hat{\varphi }}^{\mathrm{tr}}$, and $\nabla^{2}{\hat{\varphi }}^{\mathrm{tr}}$ are made through NNs’ AD it does not match the numerical spatial partial derivatives exactly, this produces various artifacts in the reconstructed conductivity. Moreover, the application of the PINN Stab-EPT optimized diffusion coefficients to the numerical calculated spatial partial derivatives also do not match and shows various artifacts in the last column of Figure A5, Figure A7 and Figure A8. The application of optimizable local coefficients to the PINN optimization in the current optimization framework is difficult without further constraints or expanding the collocation points, mainly due to an increase in the degrees of freedom of the optimization. The implementation of further constraints will be analyzed in future work.
